# Identifying key regulating miRNAs in hepatocellular carcinomas by an omics’ method

**DOI:** 10.18632/oncotarget.21865

**Published:** 2017-10-17

**Authors:** Bing He, Peng Lu, Lei Guan, Ting Li, Lei Zhang, Qing-Ge Zhu, Xiao-Xiao Ding, Shi-Ming Zhang, Xue-Mei Chen, Jing Zhao, Song Lin, Zhi-Zhen Liu, Fang-E Liu, Wang Ma, Hu-Qin Zhang

**Affiliations:** ^1^ The Key Laboratory of Biomedical Information Engineering of Ministry of Education, School of Life Science and Technology, Xi’an Jiaotong University, Xi’an 710049, P.R. China; ^2^ Gastrointestinal Surgery Department, People’s Hospital of Zhengzhou, Zhengzhou 450052, P.R. China; ^3^ Medical College, Xi’an Peihua University, Xi’an 710125, P.R. China; ^4^ Department of Oncology, The First Affiliated Hospital of Zhengzhou University, Zhengzhou 450052, P.R. China

**Keywords:** omics, miRNA, HCC

## Abstract

The miRNAs play important regulating roles in the pathogenesis of hepatocellular carcinoma (HCC). To uncover key regulating miRNAs in HCC that were neglected by traditional analyzing methods of transcriptomics data, we proposed a novel molecular-network-based omics’ (MNBO) method. With this method, we predicted HCC-regulating miRNAs, and confirmed the role of a novel miR-590-3P/*EED* axis by a clinical study and *in vitro*, *in vivo* wet-experiments. The miR-590-3P is significantly down-regulated in HCC patients. And low level of miR-590-3P in HCC is associated with poor prognosis of patients. In HCC cell lines, the miR-590-3P suppressed cell proliferation by inhibiting the transformation G1 phase to S phases of the cell cycle. Moreover, the miR-590-3P inhibited migration and invasion of HCC cells. Further investigations indicated that miR-590-3P play its roles by inhibiting polycomb protein EED. The experiments in animal model implied miR-590-3P could be a potential therapeutic agent for HCC in the future. In conclusion, the discovery of miR-590-3P revealed the MNBO would be a useful strategy to uncover key regulating miRNAs in HCC.

## INTRODUCTION

Hepatocellular carcinoma (HCC) is the second leading cause of cancer-related death of human [1, 2]. The miRNA plays important roles in regulating the pathogenesis of HCC [3]. The miRNA is small, non-coding RNA that negatively regulate the expression of target genes by mRNA degradation or translational repression [4]. Various high-throughput transcriptomics studies were performed to investigate potential key regulating miRNAs in the pathogenesis of HCC [5, 6]. Fold change and T-test were most common used methods to select candidate miRNAs in these studies. These methods would select tens to hundreds of miRNAs that may be involved in HCC. But they couldn’t indicate the importance of selected miRNAs to the pathogenesis of HCC. Therefore, it’s quite difficult to select miRNA for wet-experiments from the candidate miRNAs, and many key regulating miRNAs in HCC might be neglected in this process.

The miRNAs participate the pathogenesis of HCC by inhibiting their target genes in a complex molecular network [7-9]. It implied that we could predict the importance of a miRNA to the pathogenesis of HCC by calculating its impact on the HCC-related molecular network [8]. Therefore, we proposed a novel molecular-network-based omics’ method (MNBO) to uncover key regulating miRNAs in HCC that were neglected by traditional analyzing methods of transcriptomics data. With this method, we predicted that miR-590-3P is novel regulating miRNA in the pathogenesis of HCC. Further *in vitro* and *in vivo* experiments confirmed our prediction and found that miR-590-3P inhibits the proliferation and invasion of HCC cells by inhibiting polycomb protein EED. Moreover, the results of this study also revealed that low level of miR-590-3P is associated with poor prognosis of HCC patients and implied that miR-590-3P could be a potential therapeutic agent for HCC in the future.

## RESULTS

### Scoring the importance of miRNAs with MNBO

To score the importance of selected miRNAs to the pathogenesis of HCC, we proposed a novel molecular-network-based omics’ method (MNBO). MNBO score the importance of miRNA by calculating its ability of deregulating the molecular network in HCC (algorithm in methods). There are four key variables in MNBO: the differentially expression of miRNA, the number of its targets, the differentially expression and network importance (degree) of every target.

**Figure 1:**
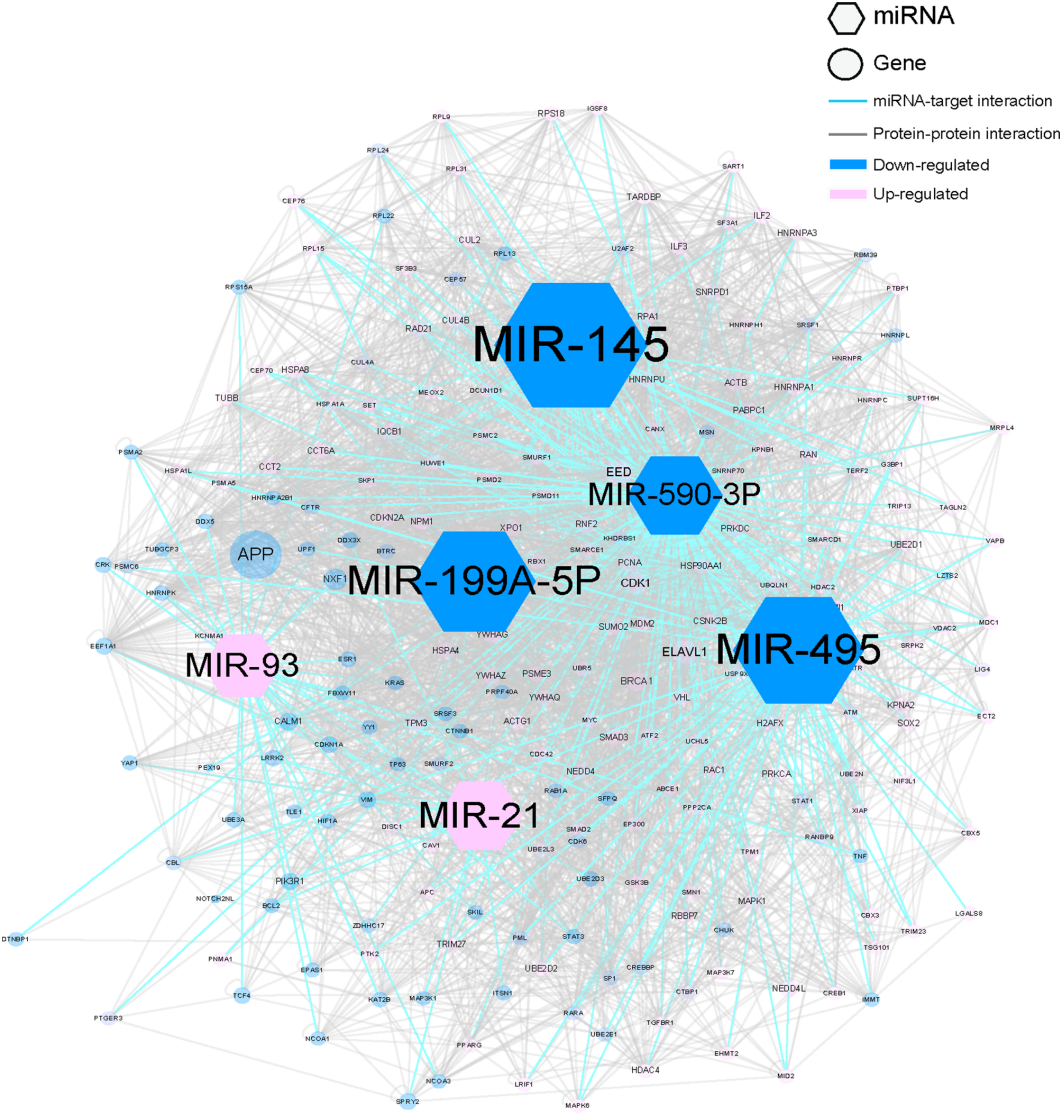
The molecular network of top 5 miRNAs according to MNBO score in HCC The size of the node represent miRNA is determined by the absolute value of MNBO score. The size of the node represent miRNA target is determined by the network influence. The larger size represents one with more importance.

To examine the ability of MNBO to discover key regulating miRNAs in HCC that were neglected by traditional analyzing methods of transcriptomics data, we downloaded transcriptomics data of 100 primary HCC tissues and matched normal liver tissues of HCC patients from GEO database (http://www.ncbi.nlm.nih.gov/gds, GSE62007) and analyzed this data with MNBO. Figure 1 showed the top five miRNAs scored by MNBO and their downstream molecular network, and the whole result is listed in Supplementary Table 1. The MNBO score not only reported key miRNA that was reported by traditional methods, like miR-21 [10], but also reported new ones, like miR-145, miR-495, miR-199A-5P and miR-590-3P (Table 1). Among these new candidates reported by MNBO, the roles of miR-145 [11, 12], miR-495 [13] and miR-199A-5P [14] in the pathogenesis of HCC have been confirmed by previous studies, while that of miR-590-3P was still ambiguous by conflicting reports [15, 16]. To examine whether miR-590-3P is a false discovery, we made further investigations on it with a larger population of HCC patients.

**Table 1:** The top five miRNAs according to MNBO score

miRNA	MNBO Score^1^	Major Target^2^
MIR-145	-5914.13	MDM2
MIR-495	-4967.78	ELAVL1
MIR-199A-5P	-4621.1	BRCA1
MIR-21	3709.772	EEF1A1
MIR-590-3P	-3466.2	EED

1. Positive MNBO score indicates the miRNA is up-regulated in HCC, while a negative one reveals the miRNA is down-regulated. The miRNA with a bigger absolute value of MNBO score is supposed to be more important than the miRNA with a smaller absolute value of MNBO score.

2. Major target is the target that has the largest network influence among all the targets of one miRNA.

### MiR-590-3P is a marker for the survival of HCC patient

**Figure 2:**
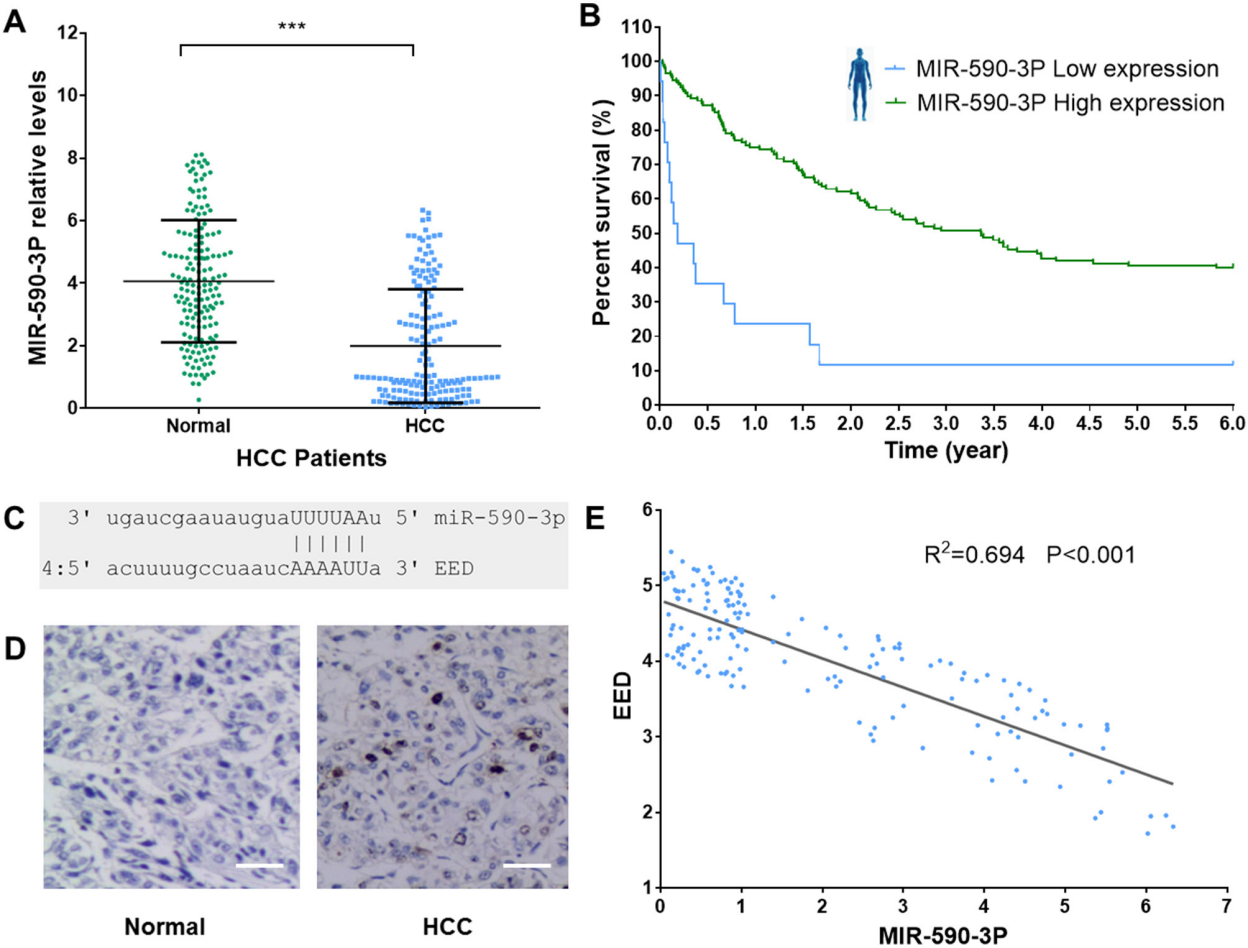
MiR-590-3P level is associated with HCC survival **(A)** MiR-590-3P relative levels in HCC tissues and adjacent normal liver tissues in 165 HCC patients; **(B)** Kaplan–Meier survival analysis of HCC patients with high (green, n=17) and low (blue, n=148) miR-590-3P levels in their surgically removed primary tumors; **(C)** the structural complementation between miR-590-3P and EED; **(D)** IHC staining for EED in HCC tissues and adjacent normal liver tissues. Scale bar, 20 μm; **(E)** the expression of miR-590-3P is significantly negatively correlated with that EED (R2=0.697, P<0.001). ^*^p<0.05, ^**^p<0.01, and ^***^p<0.001.

To investigate the role of miR-590-3P in the pathogenesis of HCC, we firstly measured its expression levels in HCC tissues and adjacent normal liver tissues in 165 HCC patients (Table 2). The result indicated that HCC tissues have significant lower miR-590-3P expression levels than adjacent normal liver tissues according to reverse transcription–quantitative PCR (RT–qPCR) measurements (Figure 2A). It’s consistent with the finding in GEO data (Figure 1).

**Table 2:** Clinic pathologic characteristics of participants

Characteristics	HCC (N=165)	Normal (N=152)
Age (mean ± standard deviation)	59.2 ± 13.2	49.5 ± 16.1
HBs Ag positive (yes/no)	95/70	56/96
HCV Ab positive (yes/no)	25/140	15/137
Tumor status (T1/T2/T3/T4)	80/60/20/5	NA
Lymph node status (N0/N1)	150/25	NA
Distant metastasis (M0/M1)	160/5	NA
TNM stage (I/II/III/IV)	75/65/20/5	NA
Histological grade (G1/G2/G3)	25/105/35	NA

HBsAg, Hepatitis B surface antigen; HCV Ab, Hepatitis C virus antibody. Tumor stage and histological grade were classified in accordance with the criteria of International Union Against Cancer (UICC)/American Joint Committee on Cancer (AJCC) 7th Edition (2009).

Prognosis is very important for HCC patients. To investigate whether miR-590-3P is a prognostic marker for patients’ survival, we performed a retrospective analysis on the data of the 165 HCC patients. The patients were divided into two groups, ‘miR-590-3P^low^’ and ‘miR-590-3P^high^’, based on the miR-590-3P expression levels measured in their surgically removed primary tumors. In this analysis, we allocated the bottom 10% (17 patients) into the miR-590-3P^low^ group and the rest (148 patients) into the miR-590-3P^high^ group to see whether miR-590-3P expression could distinguish the patients who had a substantial risk of death. The results revealed that low miR-590-3P expression was associated with a substantial risk of death in these disease-free HCC patients (Log-rank P<0.0001). The miR-590-3P^high^ group had a significantly higher survival rate than the miR-590-3P^low^ group, based on disease-free survival curves from Kaplan–Meier analysis (Figure 2B). These evidences suggested that miR-590-3P is a marker for the survival of HCC patient.

### Polycomb protein EED is a target of miR-590-3P in HCC

In MNBO method, the major target plays important roles in evaluating the importance of miRNA. The major target is the target that has the largest network influence, which is calculated by evaluating interacting neighbors and differential expressions in HCC (see methods). According to the MNBO analysis, the polycomb protein EED is the major target of miR-590-3P in HCC (Figure 1 and Table 1). To validate this prediction, we also measured EED expression levels in HCC tissues and adjacent normal liver tissues. EED protein levels were significant higher (T.test P<0.001) in HCC tissues than that in adjacent normal liver tissues according to immunohistochemistry (IHC) (Figure 2D). Moreover, the expression of *EED* is significantly negatively associated with that of miR-590-3P in 165 HCC patients (Figure 2E).

To further confirm the relation between *EED* and miR-590-3P, we transfected miR-590-3P mimics, miR-590-3P inhibitors and *EED* siRNAs into HepG2 and MHCC97H cells. At 48h after transfection, the miR-590-3P mimics and *EED* siRNAs significantly decreased EED protein levels, while miR-590-3P inhibitors significantly increased it (Figure 2A and 2C).

**Figure 3:**
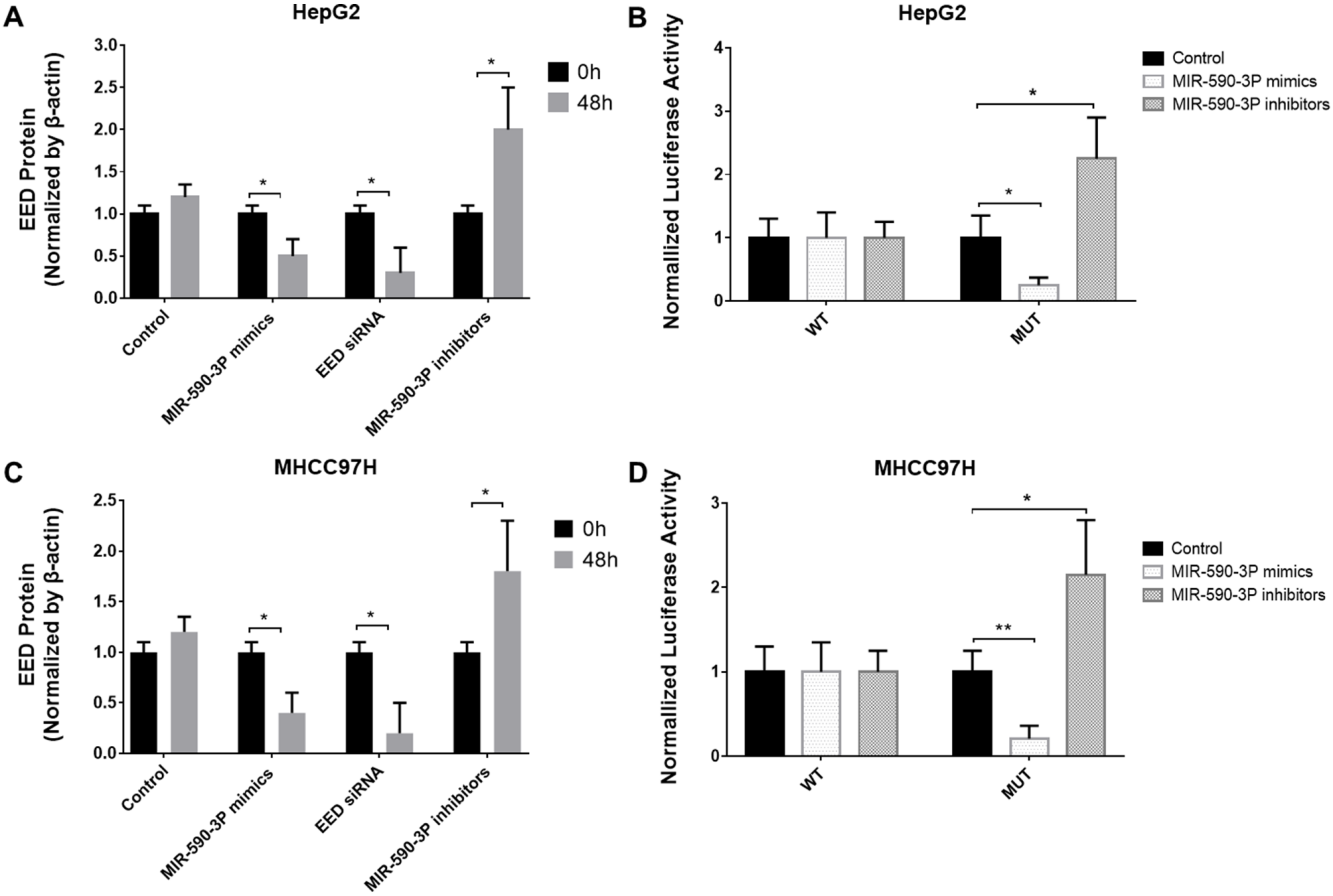
EED is a target of miR-590-3P **(A)** Protein levels of EED in HepG2 cells after transfection with miR-590-3P mimics, *EED* siRNA, miR-590-3P inhibitors or controls; **(B)** the analysis of the relative luciferase activities of *EED*-WT, *EED*-MUT in HepG2 cells after transfection with miR-590-3P mimics or miR-590-3P inhibitors; **(C)** protein levels of EED in MHCC97H cells after transfection with miR-590-3P mimics, *EED* siRNA, miR-590-3P inhibitors or controls; **(D)** the analysis of the relative luciferase activities of *EED*-WT, *EED*-MUT in MHCC97H cells after transfection with miR-590-3P mimics or miR-590-3P inhibitors; ^*^p<0.05, ^**^p<0.01, and ^***^p<0.001.

To determine whether EED is a direct target of miR-590-3P, we cloned its 3’UTR downstream of luciferase. The luciferase reporter system was then co-transfected with a miR-590-3P mimics or miR-590-3P inhibitors or an inactive control into HepG2 and MHCC97H cells. The results revealed that miR-590-3P mimics decreased the luciferase activity of the reporter gene with the wild type *EED* 3’UTR construct, but not with the mutant construct (Figure 3B and 3D). Meanwhile the miR-590-3P inhibitors increased the luciferase activity of the reporter gene with the wild type *EED* 3’UTR construct, but not with the mutant construct (Figure 3B and 3D). These evidences revealed that miR-590-3P inhibits EED by directly targeting its 3’UTR region.

### MiR-590-3P inhibited cell proliferation by blocking cell cycle

**Figure 4:**
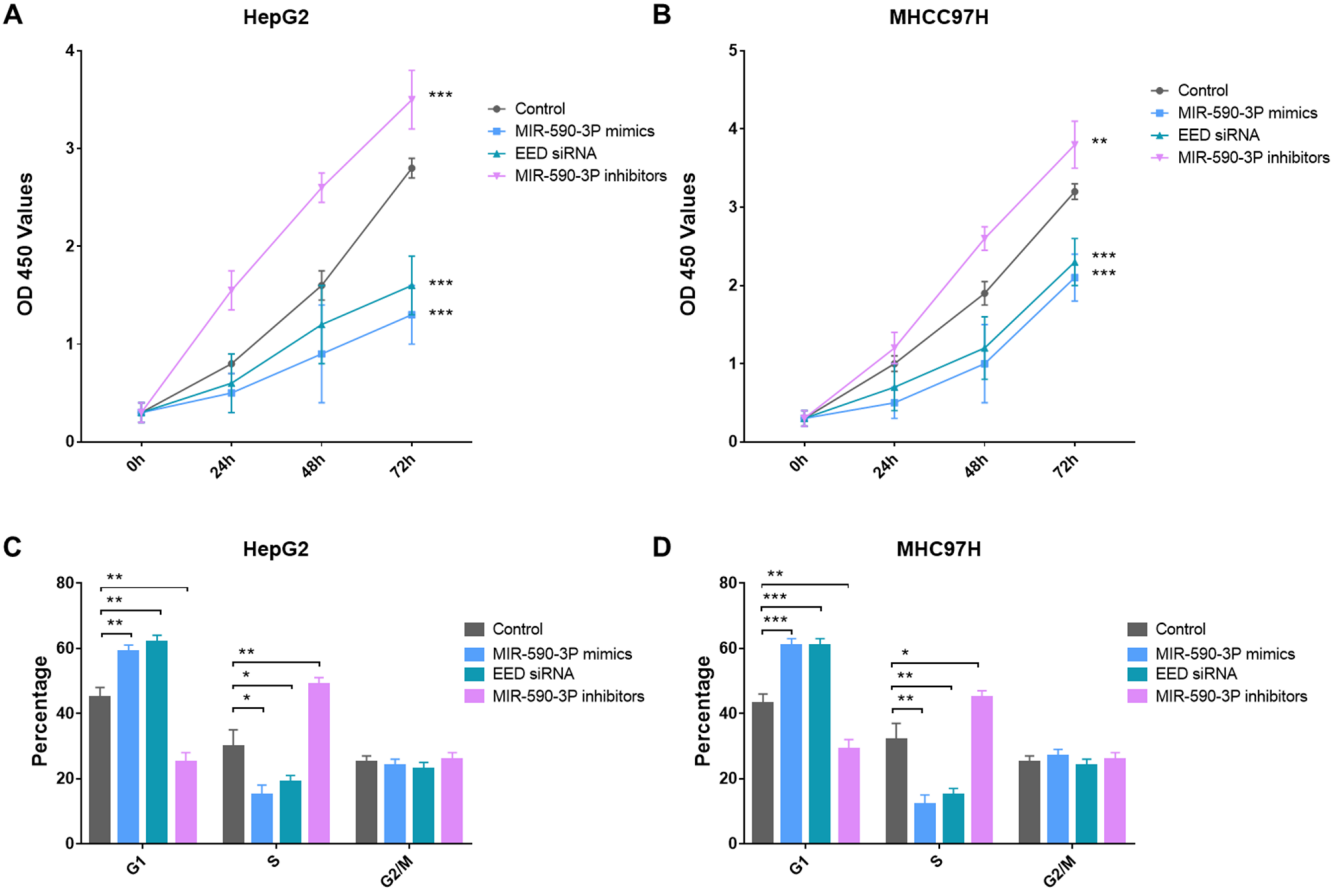
MiR-590-3P inhibits proliferation of HCC cells **(A)** Growth of HepG2 cells was shown after transfection with miR-590-3P mimics, *EED* siRNA, miR-590-3P inhibitors or controls; **(B)** growth of MHCC97H cells was shown after transfection with miR-590-3P mimics, *EED* siRNA, miR-590-3P inhibitors or controls; **(C)** cell cycle analysis of HepG2 cells after transfection with miR-590-3P mimics, *EED* siRNA, miR-590-3P inhibitors or controls; **(D)** cell cycle analysis of MHCC97H cells after transfection with miR-590-3P mimics, *EED* siRNA, miR-590-3P inhibitors or controls; the growth index as assessed at 0, 24, 48 and 72 h; ^*^p<0.05, ^**^p<0.01, and ^***^p<0.001.

Since enhanced cell proliferation is a main progress in the pathogenesis of HCC, we then investigated the potential impact of miR-590-3P and its garget *EED* on cell proliferation in HepG2 and MHCC97H HCC cell lines. HepG2 and MHCC97H cells were transfected with miR-590-3P mimics, miR-590-3P inhibitors or *EED* siRNAs. CCK8 assay indicated that the cell proliferations were significantly increased in the miR-590-3P-inhibitors-transfected HepG2 and MHCC97H cells compared with inactive-control-transfected cells (Figure 4A and 4B). In the contrast, miR-590-3P mimics *and EED* siRNAs inhibited the proliferations of the HepG2 and MHCC97H cells (Figure 2A).

To investigate how miR-590-3P inhibit cell proliferation, cell cycle analysis was then performed in HepG2 and MHCC97H cells were transfected with miR-590-3P mimics, miR-590-3P inhibitors and *EED* siRNAs. Flow cytometric analysis showed that the percentage of miR-590-3P-mimics-transfected and *EED*-siRNAs-transfected cells at G1 phase significantly increased comparing to control cells (Figure 4C and 4D). This phenomenon was associated with a concomitant decrease of cells at the S phases of the cell cycle (Figure 4C and 4D). Moreover, the percentage of miR-590-3P-inhibitors-transfected at G1 phase decreased comparing to control cells. And it was associated with a concomitant increase of cells at the S phases of the cell cycle (Figure 4C and 4D). These evidences suggested that miR-590-3P blocks the cell cycle progress and inhibits cell proliferation via *EED*.

### MiR-590-3P inhibited cell migration and invasion

**Figure 5:**
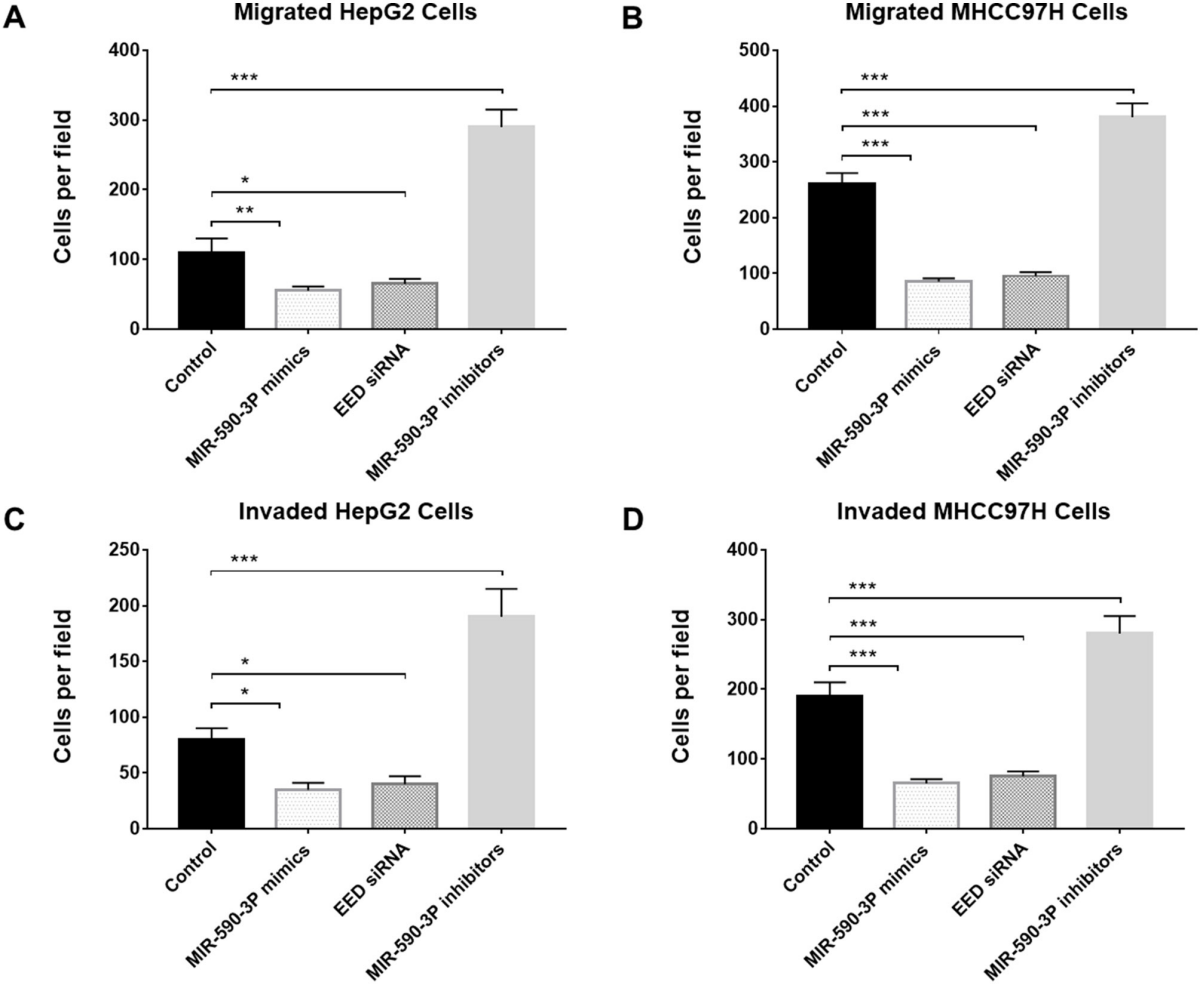
MiR-590-3P inhibits migration and invasion of HCC cells **(A)** Transwell migration analysis of HepG2 cells after transfection with miR-590-3P mimics, *EED* siRNA, miR-590-3P inhibitors or controls; **(B)** Transwell migration analysis of MHCC97H cells after transfection with miR-590-3P mimics, *EED* siRNA, miR-590-3P inhibitors or controls; **(C)** Transwell invasion analysis of HepG2 cells after transfection with miR-590-3P mimics, *EED* siRNA, miR-590-3P inhibitors or controls; **(D)** Transwell invasion analysis of MHCC97H cells after transfection with miR-590-3P mimics, *EED* siRNA, miR-590-3P inhibitors or controls; ^*^p<0.05, ^**^p<0.01, and ^***^p<0.001.

Cell migration and invasion are key progresses in tumor metastasis, which is the main reason for poor survival of HCC patients [17]. Therefore, we investigated the potential impact of miR-590-3P and its garget *EED* on cell migration and invasion by transfecting miR-590-3P mimics, miR-590-3P inhibitors or *EED* siRNAs into HepG2 and MHCC97H cells. Transwell migration assay and Matrigel invasion assay revealed that the cell migration and invasion were inhibited in miR-590-3P-mimics-transfected and *EED*-siRNAs-transfected cells compared with inactive-control-transfected cells (Figure 5). In the contrast, miR-590-3P inhibitors promoted the migration and invasion of the HepG2 and MHCC97H cells (Figure 5). These evidences suggested that miR-590-3P inhibits cell migration and invasion via *EED*.

### MiR-590-3P inhibited the tumor growth of the HepG2 xenograft in nude mice

**Figure 6:**
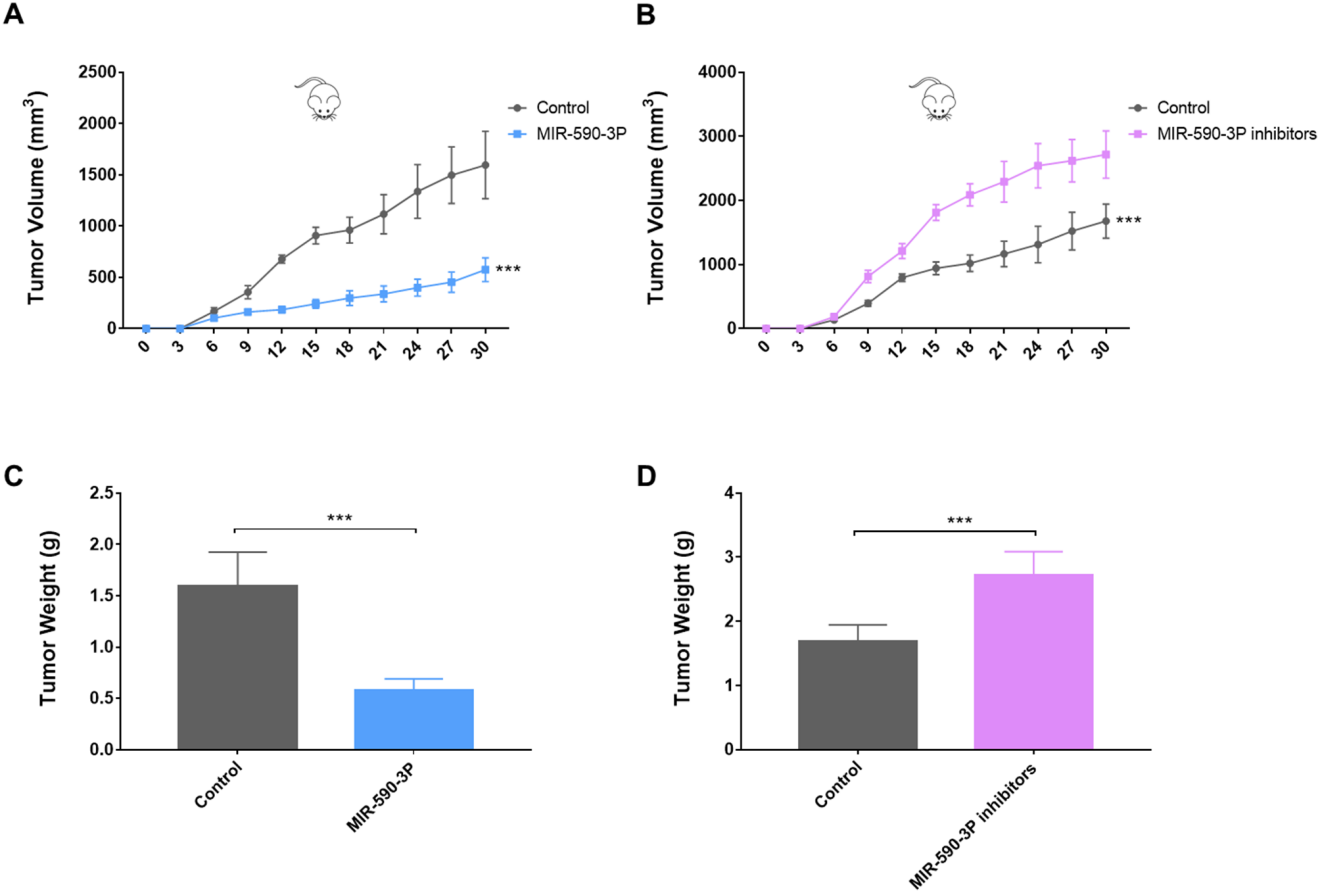
MiR-590-3P suppresses the tumor growth of the HepG2 cell xenograft in nude mice **(A)** Growth curves of xenograft in nude mice after the injection of HepG2 cells stably expressing miR-590-3P and controls; **(B)** growth curves of xenograft in nude mice after the injection of HepG2 cells stably expressing miR-590-3P inhibitors and controls; **(C)** quantification of tumor weights of xenograft tumors in mice injection of HepG2 cells stably expressing miR-590-3P and controls; **(D)** quantification of tumor weights of xenograft tumors in mice injection of HepG2 cells stably expressing miR-590-3P inhibitors and controls;. ^*^p<0.05, ^**^p<0.01, and ^***^p<0.001.

To investigate the relationship between miR-590-3P and tumorigenesis *in vivo*, the xenograft model of HepG2 cells in nude mice was adopted. We used lentiviral vectors for the stable expression of miR-590-3P and miR-590-3P inhibitor in HepG2 cells. HepG2 cells that stably expressed miR-590-3P, miR-590-3P inhibitors, or their controls were injected subcutaneously into each flank of nude mice. The tumor weights and volumes were monitored. Then the growth curves of the tumors were plotted accordingly. We found that miR-590-3P overexpression significantly decreased tumor growth and miR-590-3P inhibitors promoted it *in vivo* (Figure 6). These *in vivo* observations confirmed the key role of miR-590-3P in the tumor growth of HCC and may serve as a potential therapeutic agent for HCC in the future.

## DISCUSSION

Hepatocellular carcinoma (HCC) is the second leading cause of cancer-related death worldwide [1, 2]. The miRNA, which is small non-coding RNA, plays important roles in regulating the pathogenesis of HCC [3]. To uncover key regulating miRNAs in HCC that were neglected by traditional analyzing methods of transcriptomics data, we proposed a novel molecular-network-based omics’ (MNBO) method. According to this method, positive MNBO score indicates the miRNA is up-regulated in HCC, while a negative one reveals the miRNA is down-regulated. The absolute value of MNBO score represent the importance of miRNA in HCC. In other words, the miRNA with a bigger absolute value of MNBO score is supposed to be more important in the pathogenesis of HCC than the miRNA with a smaller one. With this method, we analyzed a transcriptomics dataset of 100 HCC patients downloaded from GEO database. Among the top 5 miRNAs scored by MNBO, only miR-21 could be discovered by traditional methods [10], the other four miRNAs, miR-145, miR-495, miR-199A-5P and miR-590-3P, were new candidates discovered by MNBO (Table 1). The regulating roles of miR-145 [11, 12], miR-495 [13] and miR-199A-5P [14] in the pathogenesis of HCC have been confirmed by previous studies, while that of miR-590-3P was still ambiguous by two conflicting reports [15, 16]. To examine whether miR-590-3P is a false discovery, we made further investigations on it.

The study in an independent group of 165 HCC patients revealed that miR-590-3P is down-regulated in HCC and is a marker for the survival of HCC patient. Moreover, expressions of the predicted major target *EED* were significantly negatively correlated with those of miR-590-3P in HCC patients (Figure 2). To investigate the potential mechanisms underlying these phenomena, more experiments were performed in HepG2 and MHCC97H HCC cell lines. The *in vitro* results indicated that miR-590-3P blocks G1/S transformation in cell cycle to inhibit cell proliferation by targeting the 3’UTR of *EED* (Figure 3 and 4). Moreover, miR-590-3P also inhibited invasion and migration of HepG2 and MHCC97H cells via *EED* (Figure 5). Further *in vivo* experiments confirmed the key regulating role of miR-590-3P in the tumor growth of HCC and implied it could be a potential therapeutic agent for HCC (Figure 6).

The miR-590-3P suppresses malignant biological behaviors of glioblastoma stem cells [18] and cancer cell migration, invasion and epithelial-mesenchymal transition in glioblastoma [19]. In this study, we found miR-590-3P inhibits cell cycle, proliferation, invasion and migration via *EED in vitro*, and suppresses tumor growth *in vivo*. *EED* encodes a polycomb protein that regulates epithelial-mesenchymal transition of cancer cells induced by TGF-β [20, 21]. Cell invasion and migration, as well as epithelial-mesenchymal transition are key progresses in tumor metastasis [17], which is leading reason for death of HCC patients [22]. It may partially explain the mechanisms of why low expression of miR-590-3P is associated with poor survival of HCC patients.

In general, our study highlights the regulating role of miR-590-3P/*EED* axis in the pathogenesis of HCC. Therefore, the MNBO method indeed can uncover key regulating miRNAs in HCC that were neglected by traditional analyzing methods of transcriptomics data. This method will be a useful tool for selecting candidate miRNAs for wet-experiments in future cancer studies.

## MATERIALS AND METHODS

### Transcriptomic data collection

The transcriptomic data of HCC refers to the data of genes and miRNAs expression in HCC and normal control samples that collected from GEO database (http://www.ncbi.nlm.nih.gov/gds, GSE62007). After quality control, HCC samples and match health controls from 100 HCC patients were used for gene and miRNA expression analysis with limma package on R platform.

### Construction of the molecular network in HCC

The molecular network used in this study was constructed by two parts: interactions between miRNA and gene during the transcription, and interactions among transcription products of genes. Experimentally validated miRNA-target interactions were collected from miRTarBase database [23] (Release 6.0). More miRNA-target interactions were predicted using DIANA [24], miRanda [25], miRDB [26], and TargetScan [27]. The miRNA-target interactions supported by at least one wet-experiment method or two prediction methods were included into the selection process using expression pattern. Since miRNAs target genes and inhibit their expression, the expression pattern of a miRNA and its target gene should be opposite. Therefore, for up-regulated miRNAs in HCC, down-regulated target genes were selected, while for down-regulated miRNAs, up-regulated target genes were selected. Finally, 100,623 miRNA-target gene interactions were included in the molecular network in HCC. Interactions among transcription products of the genes expressed in HCC were identified using protein-protein interaction data downloaded from the BioGRID database [28] (version 3.4.130). Interactions supported by at least one wet-experiment were selected to construct the PPI networks involved in HCC. A total of 116,527 PPI interactions were finally included in the molecular network of HCC.

### Calculation of MNBO score

According to our method, positive MNBO score indicates the miRNA is up-regulated in HCC, while a negative one reveals the miRNA is down-regulated. The miRNA with a bigger absolute value of MNBO score is supposed to be more important than the miRNA with a smaller absolute value of MNBO score. The MNBO Score of the miRNA in HCC is calculated by our own script written in Perl. The algorithm is described as follows:

$$\text{MNBO}=\log_{2}^{\frac{E_{C}}{E_{H}}}\sum_{\text{i}=1}^{{\text{n}}_{\text{T}}}\log_{2}^{\frac{E_{Ci}}{E_{Hi}}}D_{i}$$

$n_{\text{T}}$ is the number of all targets of a miRNA in HCC.

$E_{\text{C}}$ is the average expression value of a miRNA in HCC patients.

$E_{\text{H}}$ is the average expression value of a miRNA in matched healthy controls.

$E_{\text{Ci}}$ is the average expression value of target i in HCC patients.

$E_{\text{Hi}}$ is the average expression value of target i in matched healthy controls.

$D_{\text{i}}$ is the degree of target i in the molecular network of HCC. It’s the sum of interacting neighbors of i in the network.

The network influence of the target is calculated by $\log_{2}^{\frac{E_{C\text{i}}}{E_{H\text{i}}}}D_{i}$.

### Clinical HCC samples

Paired frozen HCC tissues and adjacent normal liver tissues were collected from HCC patients who underwent surgery at the People’s Hospital of Zhengzhou and the First Affiliated Hospital of Zhengzhou University (Table 2). A total of 165 patients with HCC were enrolled. The study was conducted in accordance with the ethical principles of the Declaration of Helsinki and Good Clinical Practice guidelines and was approved by the Ethics Committee in People’s Hospital of Zhengzhou. Informed consents were obtained from all participants.

### Kaplan–Meier survival analysis

Survival of HCC patients were analyzed by tracking 165 patients. The patients were divided into two groups, ‘MiR-590-3P ^low^’ and ‘MiR-590-3P ^high^’, based on the MiR-590-3P expression levels measured in their surgically removed primary tumors. The expression of MiR-590-3P was measured by RT-qPCR. The cutoff threshold was set to separate the top 10% MiR-590-3P ^high^ patients from the 90% MiR-590-3P ^low^ patients. The disease-free survival curves were estimated using the Kaplan–Meier method and compared with Log-rank (Mantel-Cox) test.

### Real-time reverse transcription quantitative PCR (RT-qPCR)

Total RNA was extracted with Trizol reagent (Invitrogen, Beijing, China) according to the instructions. The cDNA was synthesized from total RNA with MMLV reverse transcriptase (Invitrogen, Beijing, China) and random hexamers. Real-time PCRs were performed using ABI 7300 Sequence Detection System (Life, Beijing, China). Relative quantification was determined by normalization to the amount of GAPDH. Primers used for real-time PCR were designed by Primer Express 3.0 and synthesized in Invitrogen.

### Immunohistochemistry (IHC)

IHC was performed on tissue microarray chips (Outdo, Shanghai, China). Single serial sections were made from xenograft tumor samples. The slides were probed with the mouse anti–human EED (Abcam, Beijing, China). Then the slides were incubated with HRP-conjugated goat anti–mouse secondary antibodies. The proteins were visualized in situ with DAB chromogenic substrate.

### Culture of HCC cell lines

The human HCC cell lines HepG2 and MHCC97H were obtained from the Chinese Center for Type Cultures Collections (CCTCC, Wuhan, China). The HepG2 and MHCC97H cell lines were cultured in RPMI 1640 media (Life Technologies, Beijing, China) and supplemented with 10% fetal bovine serum (FBS) (Life Technologies, Beijing, China). All the Cells were maintained at 37°C in a humidified atmosphere with 5% CO2.

### Cell transfection

HepG2 and MHCC97H cell lines were seeded in 24-well plates at 3×10^5^ cells/wells and incubated overnight. Transfection of the *EED* siRNAs, MiR-590-3P miRNA mimics, MiR-590-3P inhibitors, inactive control cel-mir-67 (Life Technologies, Beijing, China), or pMIR-Report vectors were taken using Lipofectamine 2000 transfection reagent (Life Technologies, Beijing, China) with 1μg/ml DNA plasmid or 300 nmol of miRNA, respectively. Total proteins of HepG2 and MHCC97H cells were isolated at 24h, 48h and 72h after transfection.

### Luciferase assay

HepG2 and MHCC97H cells were seeded in 24-well plates at 3x10^5^ cells/well and then incubated for 24 hours. Then the HepG2 and MHCC97H cells were co-transfected with 0.6 μg of pGL3-EED-3’UTR or pGL3-EED-3’UTR Mut plasmid, or 0.06 ng of the phRL-SV40 control vector (Promega, Beijing, China), and 100 nM MiR-590-3P mimics or MiR-590-3P inhibitors or control RNA using Lipofectamine 2000 (Invitrogen, Beijing, China). The renilla and firefly luciferase activities were measured by a dual luciferase assay (Promega, Beijing, China) at 24 h after transfections.

### Western blot

Isolated proteins from HepG2 and MHCC97H cells at 0h and 48h after transfections were separated by 12% SDS-PAGE gel and transferred onto nitrocellulose membranes (Bio-Rad, Beijing, China). Membranes were blocked with 5% non-fat milk and incubated with anti-EED antibody (Abcam, Beijing, China) or anti-β-actin antibody (Abcam, Beijing, China). After extensive washes, the secondary antibody (Abcam, Beijing, China) was added to the system. Enhanced chemiluminescence was used for detection of EED. To check the amount of proteins transferred to nitrocellulose membrane, β-actin was used as control and detected by an anti-β-actin (C4) monoclonal antibody (Abcam; Beijing, China). The relative amounts of the transferred proteins were quantified by scanning the auto-radiographic films with a gel densitometer (Bio-Rad, Shanghai, China) and normalized to the corresponding β-actin level.

### Cell proliferation assay

Cell proliferation was detected using a Cell Counting Kit-8 assay (Dojindo, Kumamoto, Japan). HepG2 and MHCC97H cells were plated in 24-well plates at 3x10^5^ cells/well. Then HCC cells were incubated at 37°C in 10% CCK-8 diluted with normal culture medium for color conversion. Proliferation rate was measured at 24h, 48h and 72h after transfection.

### Cell cycle analysis

6×10^5^ cells were synchronized by serum starvation for 24 h. Then the cells were induced to re-enter the cell cycle by changing with 10% fetal bovine serum for 24 h. floating and adherent cells were harvested and fixed using 75% ethanol overnight at 4 °C. After that, cells were incubated with RNase A for 30 min at 37 °C. Then cells were stained with propidium iodide. Cell cycle was determined by flow cytometry.

### Cell migration and invasion assay

Cell invasion and migration were detected using transwell chamber assays (Corning, Beijing, China) with or without Matrigel (Life Technologies, Beijing, China). For the determination of HepG2 and MHCC97H cell invasion, transwell chamber was placed into a 24-well plate, then coated with 30 μl Matrigel, and incubated at 37°C for 40 minutes. In transwell assay with or without Matrigel, HepG2 and MHCC97H cells were trypsinized and seeded in chambers at the density of 6x10^4^ cells/well at 48 h after the transfection. These cells were cultured in RPMI 1640 medium with 2% serum. And 600 μl of 10% FBS-1640 was added to the lower chamber. After 24 h, migrated HepG2 and MHCC97H cells were fixed in the 100% methanol for 30 minutes. Those non-migrated HepG2 and MHCC97H cells were removed by cotton swabs. After that cells on the bottom surface of the membrane were fixed and stained with the 0.1% crystal violet for 30 minutes. Images of HepG2 and MHCC97H cells were taken using a phase-contrast microscope.

### Animal handling

Balb/c athymic nude mice were kept in the animal house, which is in a 12-hour dark/light cycle. The temperature was well controlled. And the water and food were well supplied. The rats were acclimatized to the conditions of the animal house for at least seven days before they were used in experiments.

### *In vivo* tumorigenicity assay

Tumor cells (5×10^6^) were injected subcutaneously into each flank of 4-week-old male Balb/c athymic nude mice (n = 8 per group). Mice were monitored every three day and euthanized one month later. Then tumors were dissected and weighed. The tumor volume was calculated as V (volume, mm3) = 0.5×L (length, mm)×W^2^ (width, mm^2^).

### Statistical analysis

Experiments were repeated at least three times to achieve confident results. Statistical analysis was performed using R. All the data were presented as means ± S.D. When comparing measured outcomes in two independent groups, two-sided t-test was used for analyzing continuous variables, while χ^2^ test or the Fisher exact test were used for analyzing categorical variables. When comparing repeated measured outcomes in time series groups, repeated-measures ANOVA was used for analyzing the data. The linear regression was used to determine correlations between variables. Statistical analysis was considered to be significant when P. value<0.05.

## SUPPLEMENTARY MATERIALS TABLE




