# Supplementary material for: Identifying key regulating miRNAs in hepatocellular carcinomas by an omics’ method
Source: Oncotarget. 2017 Oct 17;8(61):103919–30. doi: 10.18632/oncotarget.21865 (PMC5732776; doi:10.18632/oncotarget.21865)
Supplement: Supplementary file 1 [file oncotarget-08-103919-s001.pdf]

Identifying key regulating miRNAs in hepatocellular carcinomas by an omics' method

SUPPLEMENTARY MATERIALS

Supplementary Table 1: The top 400 miRNAs according to MNBO score

See Supplementary File 1
