# Supplementary material for: Identifying key regulating miRNAs in hepatocellular carcinomas by an omics’ method
Source: Oncotarget. 2017 Oct 17;8(61):103919–30. doi: 10.18632/oncotarget.21865 (PMC5732776; doi:10.18632/oncotarget.21865)
Supplement: Supplementary file 2 [file oncotarget-08-103919-s002.docx]

**Supplementary Table 1: The top 400 miRNAs according to MNBO score**

| **miRNA** | **MNBO Score** | **Major Target** |
| --- | --- | --- |
| MIR-145 | -5914.13 | MDM2 |
| MIR-495 | -4967.78 | ELAVL1 |
| MIR-199A-5P | -4621.1 | BRCA1 |
| MIR-21 | 3709.772 | EEF1A1 |
| MIR-590-3P | -3466.2 | EED |
| MIR-93 | 3351.178 | APP |
| MIR-325-3P | -2788.9 | ELAVL1 |
| MIR-106B | 2603.755 | APP |
| MIR-200B | -2551.2 | RNF2 |
| MIR-144 | -2465 | EED |
| MIR-223 | -2435.76 | MDM2 |
| MIR-199B-5P | -2243.42 | BRCA1 |
| MIR-101 | -2144.15 | EED |
| MIR-486-5P | -2000.14 | RNF2 |
| MIR-26A | -1925.66 | YWHAZ |
| MIR-139-5P | -1871.96 | RNF2 |
| MIR-30C | -1814.21 | EED |
| MIR-340-5P | -1785.43 | SIRT7 |
| MIR-150 | -1724.09 | TP53 |
| MIR-130A | -1697.76 | RNF2 |
| MIR-30A | -1689.44 | EED |
| MIR-301B | -1635.79 | RNF2 |
| MIR-494 | 1534.207 | CUL3 |
| MIR-195 | -1529.58 | BTRC |
| MIR-200A | -1409.65 | MDM2 |
| MIR-221 | 1375.745 | ESR1 |
| MIR-33A | -1341.38 | NPM1 |
| MIR-490-3P | -1314.2 | COPS5 |
| MIR-130B | 1306.426 | CUL3 |
| MIR-520D-3P | -1293.39 | YWHAZ |
| MIR-125A-5P | -1268.92 | TP53 |
| MIR-488 | -1268.72 | PARK2 |
| MIR-212 | 1245.132 | CUL3 |
| MIR-330-5P | -1235.5 | HNRNPA1 |
| MIR-519D | -1193.13 | RNF2 |
| MIR-15B | 1166.754 | ESR1 |
| MIR-129-5P | -1166.45 | ELAVL1 |
| MIR-122 | -1147.79 | TP53 |
| MIR-142-5P | -1123.25 | SIRT7 |
| MIR-25 | 1106.817 | ESR1 |
| MIR-376C | -1088.27 | EED |
| MIR-32 | -1025.99 | RNF2 |
| MIR-497 | -1013.38 | BTRC |
| MIR-106A | 988.0945 | APP |
| MIR-10A | -969.308 | RNF2 |
| MIR-30D | 965.9568 | CAND1 |
| MIR-520E | -962.505 | YWHAZ |
| MIR-429 | -937.826 | RNF2 |
| MIR-23B | -937.421 | MDM2 |
| MIR-410 | -936.644 | TP53 |
| MIR-224 | 927.6954 | HECW2 |
| MIR-125B | -918.831 | TP53 |
| MIR-505 | -871.434 | RNF2 |
| MIR-185 | 867.9472 | APP |
| MIR-378 | -825.803 | GRB2 |
| MIR-455-3P | -824.393 | HDAC2 |
| MIR-181C | -821.809 | EED |
| MIR-24 | -810.791 | EED |
| MIR-20A | -809.498 | COPS5 |
| MIR-218 | -806.22 | TP53 |
| MIR-128 | 797.1909 | NXF1 |
| LET-7A | -796.963 | TP53 |
| LET-7C | -788.867 | TP53 |
| MIR-425 | 778.4922 | KRAS |
| MIR-146A | -777.741 | BRCA1 |
| MIR-155 | -776.399 | EED |
| MIR-22 | -721.571 | TP53 |
| MIR-451 | -715.311 | HNRNPA1 |
| MIR-448 | -705.061 | HNRNPA1 |
| MIR-141 | -698.502 | RNF2 |
| MIR-873 | -698.415 | EED |
| MIR-31 | -672.283 | EED |
| LET-7B | -663.512 | TP53 |
| MIR-19B | -661.628 | TP53 |
| MIR-7 | -658.147 | EED |
| MIR-23A | -651.715 | MDM2 |
| MIR-335 | -651.196 | HNRNPA1 |
| MIR-124 | -639.184 | GRB2 |
| MIR-320A | -636.106 | EED |
| MIR-374A | -630.013 | RNF2 |
| MIR-876-5P | -612.71 | ELAVL1 |
| MIR-361-5P | 609.3889 | CUL5 |
| MIR-29C | -609 | MDM2 |
| MIR-422A | -602.03 | GRB2 |
| MIR-153 | -600.475 | BRCA1 |
| MIR-9 | -592.395 | ELAVL1 |
| LET-7E | -592.271 | TP53 |
| MIR-491-5P | -591.908 | TP53 |
| MIR-190B | -574.354 | BRCA1 |
| MIR-1 | -553.288 | BRCA1 |
| MIR-342-3P | -546.652 | EP300 |
| MIR-15A | 543.3046 | ESR1 |
| MIR-143 | 542.2806 | NXF1 |
| MIR-377 | -541.303 | EED |
| MIR-371-5P | 523.2846 | CUL3 |
| MIR-590-5P | -519.808 | RNF2 |
| MIR-520A-3P | -516.974 | YWHAZ |
| MIR-181D | -510.604 | EED |
| MIR-216A | 510.0781 | ESR1 |
| MIR-140-5P | -506.461 | RNF2 |
| MIR-320D | -505.237 | BRCA1 |
| MIR-362-3P | -500.107 | EED |
| MIR-296-3P | -494.504 | TP53 |
| MIR-320C | 489.3721 | FN1 |
| MIR-653 | -481.656 | NPM1 |
| MIR-1297 | -466.738 | YWHAZ |
| MIR-154 | -466.586 | ILF3 |
| MIR-449B | -463.297 | BRCA1 |
| MIR-381 | 460.6728 | APP |
| MIR-181A | -456.276 | EED |
| MIR-148A | 455.6164 | EGFR |
| MIR-136 | -451.684 | RNF2 |
| MIR-449A | -449.877 | BRCA1 |
| MIR-503 | -420.16 | EED |
| MIR-217 | -415.566 | HNRNPA1 |
| MIR-373 | -407.857 | HSP90AA1 |
| MIR-96 | 404.4777 | FN1 |
| MIR-485-5P | -400.527 | TP53 |
| MIR-302C | -398.743 | MDM2 |
| MIR-504 | -398.656 | TP53 |
| MIR-219-5P | -389.836 | RNF2 |
| MIR-382 | -387.291 | HNRNPA1 |
| MIR-138 | -386.266 | SMURF1 |
| MIR-302A | -385.92 | MDM2 |
| MIR-34A | 380.5093 | SIRT1 |
| MIR-151-3P | 378.0115 | APP |
| MIR-367 | -376.843 | RNF2 |
| MIR-339-5P | -376.755 | RNF2 |
| MIR-186 | 375.1786 | APP |
| MIR-148B | 372.4627 | EGFR |
| MIR-489 | -368.737 | HDAC2 |
| MIR-18A | 367.3461 | EGFR |
| MIR-107 | 362.8327 | EGFR |
| MIR-543-3P | -362.167 | RNF2 |
| MIR-384-3P | -358.039 | HNRNPA1 |
| MIR-383 | 356.6347 | APP |
| MIR-542-3P | -354.314 | RNF2 |
| MIR-27A | -354.236 | ELAVL1 |
| MIR-206 | -352.879 | BRCA1 |
| MIR-129-3P | -344.97 | BAG3 |
| MIR-1224-5P | -342.494 | YWHAZ |
| MIR-338-3P | -341.614 | TP53 |
| MIR-181B | -339.112 | EED |
| MIR-409-3P | -337.759 | CREBBP |
| MIR-544 | -336.84 | YWHAZ |
| MIR-299-3P | -335.116 | POU5F1 |
| MIR-135A | -330.11 | HNRNPA1 |
| MIR-193B | 329.8293 | ESR1 |
| MIR-222 | 329.7292 | ESR1 |
| MIR-26B | -328.653 | YWHAZ |
| MIR-137 | -328.642 | HNRNPA1 |
| MIR-320B | 319.317 | FN1 |
| MIR-20B | -314.695 | COPS5 |
| MIR-142-3P | -312.443 | TARDBP |
| MIR-149 | -312.226 | TP53 |
| MIR-655 | -312.2 | YWHAZ |
| LET-7G | -310.394 | TP53 |
| MIR-18B | 305.7706 | EGFR |
| MIR-190 | -302.86 | BRCA1 |
| MIR-363 | -293.533 | EED |
| MIR-29B | 293.419 | EEF1A1 |
| MIR-506 | -291.299 | HDAC5 |
| MIR-376A | -284.412 | RNF2 |
| MIR-211 | -275.843 | RNF2 |
| MIR-875-5P | -269.765 | RNF2 |
| MIR-214 | -267.078 | EED |
| MIR-16 | -265.031 | BTRC |
| MIR-99A | -248.743 | RNF2 |
| MIR-33B | 246.9327 | EGFR |
| MIR-1271 | 244.1638 | KRAS |
| MIR-582-5P | -244.157 | HNRNPA1 |
| MIR-296-5P | -243.03 | E2F1 |
| MIR-27B | -237.866 | ELAVL1 |
| MIR-200C | -237.259 | RNF2 |
| MIR-372 | -236.084 | YWHAZ |
| MIR-346 | -235.874 | HNRNPA1 |
| MIR-29A | -233.739 | MDM2 |
| LET-7F | -229.035 | TP53 |
| MIR-532-3P | -227.41 | HNRNPU |
| MIR-376B | -225.133 | RNF2 |
| MIR-132 | -223.584 | BRCA1 |
| MIR-599 | -223.267 | PARK2 |
| MIR-455-5P | -221.833 | HNRNPA1 |
| MIR-758 | -220.343 | MDM2 |
| MIR-499-5P | -218.081 | EED |
| MIR-135B | -215.158 | HNRNPA1 |
| MIR-369-3P | -209.614 | EP300 |
| MIR-370 | -208.804 | HNRNPA1 |
| MIR-302E | -205.37 | YWHAZ |
| MIR-409-5P | -203.968 | UBE2L3 |
| MIR-152 | -203.136 | SIRT7 |
| MIR-133A | -202.85 | UBQLN1 |
| MIR-379 | -202.261 | TP53 |
| MIR-708 | -197.681 | YWHAZ |
| MIR-411 | -196.65 | EED |
| MIR-520D-5P | -195.923 | EED |
| MIR-204 | -195.324 | RNF2 |
| MIR-375 | -193.028 | YWHAZ |
| MIR-324-5P | 182.0313 | APP |
| MIR-613 | -180.852 | BRCA1 |
| MIR-140-3P | 167.6967 | CAND1 |
| LET-7D | -165.443 | TP53 |
| MIR-196A | -164.169 | CUL4B |
| MIR-208A | -160.148 | ILF3 |
| MIR-938 | -156.921 | EED |
| MIR-17 | 151.4208 | APP |
| MIR-202-5P | -145.939 | RNF2 |
| MIR-216B | 140.4046 | ESR1 |
| MIR-493 | -138.834 | HNRNPU |
| MIR-34C-5P | -135.978 | BRCA1 |
| MIR-196B | -131.378 | CUL4B |
| MIR-331-3P | 127.9527 | ARRB1 |
| MIR-323-3P | -122.205 | SMURF1 |
| MIR-299-5P | -118.61 | VCP |
| MIR-133B | -117.568 | UBQLN1 |
| MIR-421-3P | 117.1109 | CUL3 |
| MIR-146B-5P | 116.2487 | WWOX |
| MIR-183 | -114.443 | EED |
| MIR-431 | -114.158 | TP53 |
| MIR-92B | 111.139 | ESR1 |
| MIR-302D | -109.765 | MDM2 |
| MIR-365 | -109.494 | HSPA8 |
| MIR-191 | 108.5743 | CALM1 |
| MIR-187 | -105.416 | PARK2 |
| MIR-10B | 103.4358 | EGFR |
| MIR-302B | -101.268 | MDM2 |
| MIR-28-3P | -91.6965 | FAF2 |
| MIR-30E | -88.0702 | EED |
| MIR-330-3P | -87.9108 | BMI1 |
| MIR-92A | 86.13896 | ESR1 |
| MIR-208B | -85.1481 | ILF3 |
| MIR-215 | -84.3292 | CCT2 |
| MIR-1251 | -82.8721 | CBX5 |
| MIR-125A-3P | -81.3023 | BRCA1 |
| MIR-192 | -80.6617 | CCT2 |
| MIR-210 | 80.29941 | RPL22 |
| MIR-1184 | -75.0012 | ILF3 |
| MIR-203 | 69.73691 | EGFR |
| MIR-329 | -68.173 | EED |
| MIR-665 | 67.98174 | NXF1 |
| MIR-100 | -67.956 | PPIA |
| MIR-19A | 63.19192 | CAND1 |
| MIR-98 | -62.2539 | TP53 |
| MIR-193A-3P | 60.72501 | ESR1 |
| MIR-668 | -58.7316 | HNRNPA1 |
| MIR-532-5P | -53.2663 | SMAD2 |
| MIR-28-5P | -52.8577 | PARK2 |
| MIR-326 | -52.4873 | HNRNPA1 |
| MIR-194 | 50.3546 | APP |
| MIR-1298 | -49.8275 | PPP2CA |
| MIR-184 | -45.2528 | ZDHHC17 |
| MIR-762 | 43.53953 | RBPMS |
| MIR-505-3P | -42.4521 | ILF2 |
| MIR-30B | 41.81356 | CAND1 |
| MIR-487B | 37.4912 | TP63 |
| MIR-188-5P | -37.0299 | EED |
| MIR-1197 | 36.73061 | XRCC5 |
| MIR-452 | -35.4537 | RPA1 |
| MIR-205 | 33.39468 | CAND1 |
| MIR-301A | -33.386 | RNF2 |
| MIR-197 | 33.01562 | SF3B1 |
| MIR-874 | 32.7755 | ESR1 |
| MIR-433-3P | -30.832 | HNRNPA1 |
| MIR-99B | -30.7145 | RNF2 |
| MIR-934 | -29.5279 | SKP1 |
| MIR-126-3P | -27.107 | GBP2 |
| MIR-511-5P | -27.0059 | SKP1 |
| MIR-559 | -26.0524 | ZRANB2 |
| MIR-1286 | -24.1875 | CDKN2A |
| MIR-892A | -23.1794 | TRIM23 |
| MIR-362-5P | -23.1143 | DCUN1D1 |
| MIR-520H | -22.8432 | MAPK9 |
| MIR-423-5P | 22.68834 | AP2M1 |
| MIR-548C-5P | -21.9833 | ZRANB2 |
| MIR-615-3P | -21.5465 | RUVBL1 |
| MIR-331-5P | -21.4511 | PSMD10 |
| MIR-509-3-5P | -20.9351 | NAP1L1 |
| LET-7I | -19.6329 | TP53 |
| MIR-606 | -19.3993 | ZRANB2 |
| MIR-1257 | -18.6375 | CDKN2A |
| MIR-328-3P | 18.3276 | CRK |
| MIR-875-3P | -18.1795 | SKP1 |
| MIR-219-1-3P | 17.78107 | GHR |
| MIR-126-5P | -17.5814 | MAP3K7 |
| MIR-423-3P | -17.4497 | PABPC1 |
| MIR-548C-3P | -17.3822 | MAP3K7 |
| MIR-940 | -16.2936 | CDKN2A |
| MIR-541-3P | -16.0659 | MAPK9 |
| MIR-486-3P | -16.0561 | PNKD |
| MIR-188-3P | 15.87194 | UBE2D3 |
| MIR-519B-3P | -15.5898 | MAP4 |
| MIR-509-5P | -15.3149 | NAP1L1 |
| MIR-519C-3P | -14.943 | MAP4 |
| MIR-654-5P | -14.7756 | MAPK9 |
| MIR-548I | -13.9157 | ZRANB2 |
| MIR-491-3P | -13.6349 | TRIM23 |
| MIR-487A | 13.29834 | HECW2 |
| MIR-548P | -13.2762 | TRIM23 |
| MIR-548D-3P | -12.5007 | MAP3K7 |
| MIR-761 | -12.4412 | DNM1 |
| MIR-922 | -12.4048 | ZRANB2 |
| MIR-147B | -11.9551 | UBE2N |
| MIR-1254 | -11.9273 | PML |
| MIR-513A-3P | -11.9192 | DAZAP1 |
| MIR-1205 | -11.6348 | MAP3K7 |
| MIR-920 | -11.6326 | MAP4 |
| MIR-1294 | -11.5671 | TP53 |
| MIR-507 | -10.8491 | RCOR3 |
| MIR-202-3P | -10.6804 | NAP1L1 |
| MIR-520A-5P | -10.5645 | PML |
| MIR-607 | -10.4368 | MAP3K7 |
| MIR-541-5P | -10.3141 | NAP1L1 |
| MIR-302F | 10.17881 | CTNNB1 |
| MIR-524-5P | -10.1226 | EED |
| MIR-483-3P | -10.05 | RCOR3 |
| MIR-1539 | 9.98697 | NFIA |
| MIR-1179 | -9.86114 | TRIM23 |
| MIR-513A-5P | 9.767813 | KRAS |
| MIR-548K | 9.755351 | RBPMS |
| MIR-361-3P | -9.44165 | KDM5C |
| MIR-298 | -9.4183 | POGZ |
| MIR-1224-3P | -9.37622 | PIP4K2C |
| MIR-1278 | -9.32805 | PSMB5 |
| MIR-1323 | -9.19798 | MAP3K7 |
| MIR-1207-5P | -9.13911 | PML |
| MIR-675-3P | -9.11771 | ZRANB2 |
| MIR-765 | -8.96335 | MAP3K7 |
| MIR-1279 | 8.523258 | NFIA |
| MIR-548B-5P | -8.52243 | ZRANB2 |
| MIR-345-3P | 7.793782 | CRK |
| MIR-450A-5P | -7.73631 | CREB1 |
| MIR-512-5P | -7.70059 | MAPK9 |
| MIR-608 | -7.59378 | MAPK9 |
| MIR-935 | -7.08903 | MAP3K7 |
| MIR-1302 | -7.06562 | SKP1 |
| MIR-744 | 6.939439 | PPP5C |
| MIR-1912 | -6.93713 | NAP1L1 |
| MIR-134 | 6.850781 | CAND1 |
| MIR-1207-3P | -6.84509 | TPM1 |
| MIR-551B | -6.70886 | CBX3 |
| MIR-646 | -6.67135 | MAP3K7 |
| MIR-1264 | -6.47204 | ITGB1 |
| MIR-630 | -6.40071 | EXO1 |
| MIR-548N | -6.32545 | ZRANB2 |
| MIR-1248 | -6.30948 | HLTF |
| MIR-641 | -6.30376 | DAZAP1 |
| MIR-588 | 5.843075 | UBE2D3 |
| MIR-548A-5P | -5.83887 | ZRANB2 |
| MIR-576-5P | -5.80876 | TRIM23 |
| MIR-582-3P | -5.77426 | MAPK9 |
| MIR-548D-5P | 5.705676 | KRAS |
| MIR-645 | -5.69166 | MAPK9 |
| MIR-1283 | 5.577596 | NXF1 |
| MIR-562 | -5.39259 | NAP1L1 |
| MIR-626 | -5.37427 | TRIM23 |
| MIR-1284 | 5.299337 | KRAS |
| MIR-575 | 5.23927 | NDEL1 |
| MIR-1208 | -5.05853 | TRIM55 |
| MIR-557 | -4.99672 | RCOR3 |
| MIR-618 | -4.98271 | UFD1L |
| MIR-558 | -4.98015 | STXBP1 |
| MIR-450B-5P | -4.97857 | TSPAN17 |
| MIR-876-3P | -4.96966 | POGZ |
| MIR-148B-5P | -4.8823 | MRRF |
| MIR-711 | 4.767712 | CARS |
| MIR-515-3P | -4.74312 | TRIM55 |
| MIR-345-5P | -4.71367 | BRMS1 |
| MIR-548B-3P | -4.68419 | SAE1 |
| MIR-324-3P | 4.599844 | PHF1 |
| MIR-592 | -4.53549 | MAP4 |
| MIR-586 | -4.49212 | KIAA1598 |
| MIR-34B-5P | -4.47499 | LAMP2 |
| MIR-1825 | 4.391582 | CD40 |
| MIR-1231 | -4.31339 | MEIS2 |
| MIR-485-3P | -4.31086 | PDCD6IP |
| MIR-323B-5P | -4.20053 | CDKN2B |
| MIR-125B-2-3P | -4.01176 | DAZAP1 |
| MIR-548A-3P | -3.97132 | NAP1L1 |
| MIR-1225-5P | -3.93479 | PLCB1 |
| MIR-1275 | -3.90845 | PTGER3 |
| MIR-636 | -3.77273 | RECQL5 |
| MIR-620 | -3.77117 | APAF1 |
| MIR-380-5P | -3.7405 | PPM1B |
| MIR-649 | -3.59589 | POGZ |
| MIR-1270 | -3.55061 | APAF1 |
| MIR-380-3P | -3.38531 | MAP3K7 |
| MIR-502-3P | -3.32254 | CDK6 |
| MIR-628-5P | -3.28097 | MAPK9 |
| MIR-297 | -3.27779 | PTGER3 |
| MIR-1276 | -3.23087 | RNF7 |
| MIR-556-3P | 3.165062 | TP63 |
| MIR-1249 | -3.10448 | EPHB2 |
| MIR-578 | -2.97405 | SRP9 |
| MIR-587 | -2.91796 | ZRANB2 |
| MIR-654-3P | -2.82978 | FXR1 |
| MIR-621 | -2.818 | LEF1 |
| MIR-501-5P | -2.81696 | NAP1L1 |
| MIR-568 | -2.79884 | H2AFV |
| MIR-484 | -2.77422 | SAE1 |

1. Positive MNBO score indicates the miRNA is up-regulated in HCC, while a negative one reveals the miRNA is down-regulated. The miRNA with a bigger absolute value of MNBO score is supposed to be more important than the miRNA with a smaller absolute value of MNBO score.
2. Major target is the target that has the largest network influence among all the targets of one miRNA.
